# Knowledge, Attitudes, and Practices of Nurses Regarding Needle Stick Injuries, HIV, and Hepatitis B Prevention in a Tertiary Care Center in Nagpur, India: A Cross-Sectional Study

**DOI:** 10.7759/cureus.87160

**Published:** 2025-07-02

**Authors:** Sanjay Sain, Nishaant Ramasamy, Ishan Verma, Jaya Prasad Tripathy, Udit Narang, Sunita Kumbhalkar, Amol H Dube, Keshao B Nagpure

**Affiliations:** 1 General Medicine, All India Institute of Medical Sciences, Nagpur, Nagpur, IND; 2 Community Medicine, All India Institute of Medical Sciences, Nagpur, Nagpur, IND; 3 Preventive Medicine, Jawaharlal Institute of Postgraduate Medical Education and Research (JIPMER), Puducherry, IND; 4 General Medicine, LN Medical College and Research Center, Bhopal, IND; 5 General Medicine: Cardiology, All India Institute of Medical Sciences, Nagpur, Nagpur, IND

**Keywords:** healthcare workers, hepatitis b prevention, hiv prevention, india, infection control, needlestick injuries, nurses, occupational exposure, post-exposure prophylaxis, tertiary care centre

## Abstract

Introduction

Needlestick injuries (NSIs) pose serious health risks to healthcare workers, especially nurses, due to the potential transmission of HIV and Hepatitis B. Despite available guidelines for vaccination and post-exposure prophylaxis (PEP), awareness and adherence remain low, particularly in developing countries like India. Nurses face high NSI risk due to their frontline role in patient care, yet limited Indian studies assess their knowledge, attitudes, and practices (KAP) on this issue. This cross-sectional study was conducted among nurses at a tertiary care hospital in Nagpur using a validated questionnaire to evaluate KAP regarding NSI prevention and management. The findings aim to highlight existing gaps and support targeted interventions to reduce NSI-related infections and improve occupational safety among nurses.

Methods

A cross-sectional analytical study was conducted at a tertiary care hospital in Nagpur City, situated in the central part of India, from December 2023 to February 2024. Based on an assumed 50% prevalence, the calculated sample size was 216 nurses. A validated, self-administered online questionnaire assessing KAP related to needlestick injuries, HIV, and Hepatitis B was distributed via Google Forms (Google, Mountain View, California, US) to 500 nurses; 227 responded. Responses were scored and categorized into good, average, or poor knowledge. Data were analysed using Stata version 18 (StataCorp LLC, College Station, TX, US) with appropriate statistical tests. Ethical approval was obtained for the study.

Results

The prevalence of NSIs was 40.5% (92 out of 227 participants; 95% CI: 34.1%-47.2%). Good knowledge was observed in 203 (89.4%) for NSI, 159 (70%) for HIV, and 211 (93%) participants for Hepatitis B. However, 68 participants (30%) had only average or poor knowledge of HIV. No significant association was found between NSI occurrence and age, gender, experience, or marital status. Similarly, knowledge levels for NSI, HIV, and Hepatitis B were not significantly influenced by demographic factors. Attitudes were largely positive: 203 participants (92.6%) perceived the NSI risk as serious, and 214 (97.7%) believed NSIs are preventable. Most supported immediate reporting and PEP for HIV (204; 94.4%) and Hep B (201; 93%). Despite this, only 27 of 87 (31%) received PEP after injury. NSIs frequently involved hypodermic needles or lancets and occurred during disposal or recapping. However, unsafe practices persisted, with 53 of 219 (24%) of nurses recapping needles, a behaviour significantly associated with NSI incidence (p = 0.045). The most cited barriers to NSI reporting were time constraints (166; 78.3%) and lack of awareness about reporting procedures (98; 46%).

Conclusion

This study highlights that nurses possess good knowledge and positive attitudes toward NSI, HIV, and Hepatitis B. However, gaps remain in translating knowledge into safe practices and consistent PEP adherence. Strengthening training, reinforcing safety protocols, and fostering a supportive institutional environment are essential to reduce occupational risks and enhance healthcare worker safety.

## Introduction

Needlestick injuries (NSI) are a major occupational hazard among healthcare workers (HCWs), particularly nurses, due to the risk of transmission of bloodborne infections such as Hepatitis B virus (HBV), Hepatitis C virus (HCV), and Human Immunodeficiency Virus (HIV). According to the Centers for Disease Control and Prevention (CDC), a needlestick injury occurs when a needle or other sharp object punctures the skin after contact with blood, tissue, or body fluids of another person [1]. These injuries commonly result from blood collection needles, suture needles, intravenous (IV) delivery systems, and hypodermic needles [2].

Standardized protocols exist for the prevention and post-exposure management of HBV and HIV [3-6]. The World Health Organization (WHO) estimates that needlestick injuries account for approximately 66,000 HBV, 16,000 HCV, and 200-5,000 HIV infections annually among HCWs, with percutaneous occupational exposure contributing to 4.4% of HIV, 37% of HBV, and 39% of HCV cases [7]. Despite the availability of HBV vaccination, coverage among HCWs in developing countries remains significantly lower than in developed nations [8]. Similarly, post-exposure prophylaxis (PEP) with antiretroviral therapy (ART) for 28 days has been shown to reduce HIV transmission [9]. The recommended PEP regimen includes two nucleoside reverse transcriptase inhibitors (NRTIs) combined with a third drug, typically a protease inhibitor (e.g., Tenofovir, Emtricitabine, plus either Dolutegravir or Raltegravir), and should be initiated as early as possible, ideally within 72 hours [10].

The risk of infection transmission following an NSI varies, with HBV having the highest rate (6-30%), followed by HCV (1.8%) and HIV (0.3%) [10]. Indian data on NSI remains largely underreported, and available statistics suggest that approximately 3-6 billion injections are administered annually in India, of which nearly two-thirds are unsafe [11]. The Exposure Prevention Information Network (EPINet) has reported 27 NSIs per 100 hospital beds, while Indian studies indicate a rate of 8.9 to 10.4 NSIs per 100 beds [12]. Additionally, a recent meta-analysis estimated the global prevalence of NSIs to be 44.5% [13]. Considering these factors, a low reported incidence of NSI should not be mistaken for an actual low occurrence, as underreporting remains a significant concern across healthcare settings [12]. Developing countries, including India, face higher NSI risks due to factors such as hospital overcrowding, high patient-to-HCW ratios, inadequate awareness, limited adherence to infection control protocols, and restricted access to PEP. The incidence of HBV among HCWs is reported to be four times higher than in the general population [8]. Furthermore, around 0.5% of healthcare workers worldwide contract HIV each year, highlighting the ongoing occupational risk in clinical settings [14].

As per 2022 data from the Indian Nursing Council (INC), the country has approximately 33.41 lakh registered nurses [15]. Among HCWs, nurses are particularly vulnerable due to their direct involvement in procedures such as administering injections, phlebotomy, inserting IV lines, and assisting in surgical interventions [16]. Studies indicate that nurses experience higher rates of percutaneous and mucosal exposures to blood and body fluids compared to other HCWs [17].

Efforts to mitigate NSIs have been implemented in developed nations, such as the Needlestick Safety and Prevention Act enacted in the United States (1999-2000), which established guidelines for safer needle devices and enhanced infection control practices [18]. However, such legislative measures are yet to be fully realized in India and many other developing countries, where NSIs remain a significant public health concern.

There is a dearth of comprehensive studies from India that assess the knowledge, attitudes, and practices (KAP) related to NSI, Hepatitis B, and HIV collectively. This study aims to assess the KAP of nurses regarding NSI prevention and associated bloodborne infections in a tertiary care hospital in Nagpur, India. By identifying existing gaps in awareness and adherence to safety protocols, this study seeks to contribute to the development of targeted interventions aimed at reducing NSI incidence and enhancing occupational safety among nurses.

## Materials and methods

Study design and setting

This was a cross-sectional analytical study conducted at a tertiary care hospital in Nagpur, situated in the central part of India, from December 2023 to February 2024.

Sample size calculation

The sample size was determined using the single proportion formula:

$n = z_{1 - \alpha/2}^2 \left( \frac{p(1 - p)}{d^2} \right)$; at a 95% confidence interval, where $z_{1 - \alpha/2}$ = 1.96 (standard normal value for 95% CI); p = 50% (prevalence of good knowledge regarding NSI among nurses [19]); d = 7% (margin of error). A minimum sample size of 196 was calculated, and with a 10% dropout rate, the final required sample was 216 participants.

Subject selection

Inclusion criteria included nurses aged ≥18 years working in the hospital. Exclusion criteria included nurses who declined consent or were on medical leave due to illness.

Ethical considerations

The study was approved by the Institutional Ethics Committee (IEC/Pharmac/2023/694). Informed consent was obtained from all participants before study participation, and confidentiality was maintained.

Data collection

A self-administered online questionnaire was developed using validated questions from prior studies (Appendices) [7-14,16]. The self-developed questionnaire underwent face validation by experts to ensure relevance and appropriateness. This process involved experts reviewing the questionnaire's items and overall structure to assess clarity, readability, and how well the questions aligned with the study's objectives. The questionnaire, hosted on Google Forms (Google, Mountain View, California, US), was distributed to all 500 nursing officers via WhatsApp, with reminders sent every 10 days. The data collection period lasted two months, and 227 responses were obtained.

The questionnaire comprised 66 questions across four sections: Socio-demographic characteristics (6 questions), knowledge of NSI, HBV, and HIV prevention (21 questions), attitudes toward NSI and infection prevention (18 questions), and practices related to NSI prevention (7 questions).

The knowledge section was categorized separately for NSI, HIV, and HBV. Each correct response was awarded one point, while incorrect responses received zero points. For multiple-response questions, scores ranged from 1 to 3 points based on the number of correct answers. The total knowledge score for NSI, HIV, and HBV was categorized as follows, based on a validated classification system [9]: good knowledge: >75% (score >6), average knowledge: 50-75% (score 4-5) and poor knowledge: <50% (score <3).

Statistical analysis

All the data obtained from the questionnaire were entered into a Microsoft Excel sheet (Microsoft Corporation, Redmond, WA, US), and statistical analysis was done using Stata version 18 (StataCorp LLC, College Station, TX, US) [20]. The prevalence of NSI was expressed in percentage with a 95% confidence interval. The knowledge categories were summarized as frequencies and percentages. The knowledge categories were compared across socio-demographic characteristics of the participants by the t-test/Wilcoxon rank sum test and the analysis of variance (ANOVA)/Kruskal-Wallis test for continuous variables based on the number of groups and normality, whereas for categorical variables, the chi-squared/Fisher exact test was used. The knowledge category was compared between the NSI categories using the chi-squared test. The responses for the attitude domain were summarized as frequencies and percentages. The practice domain responses were summarized as frequencies and percentages and were compared between the NSI groups, and the chi-squared test was used to assess statistical significance.

## Results

**Figure 1:**
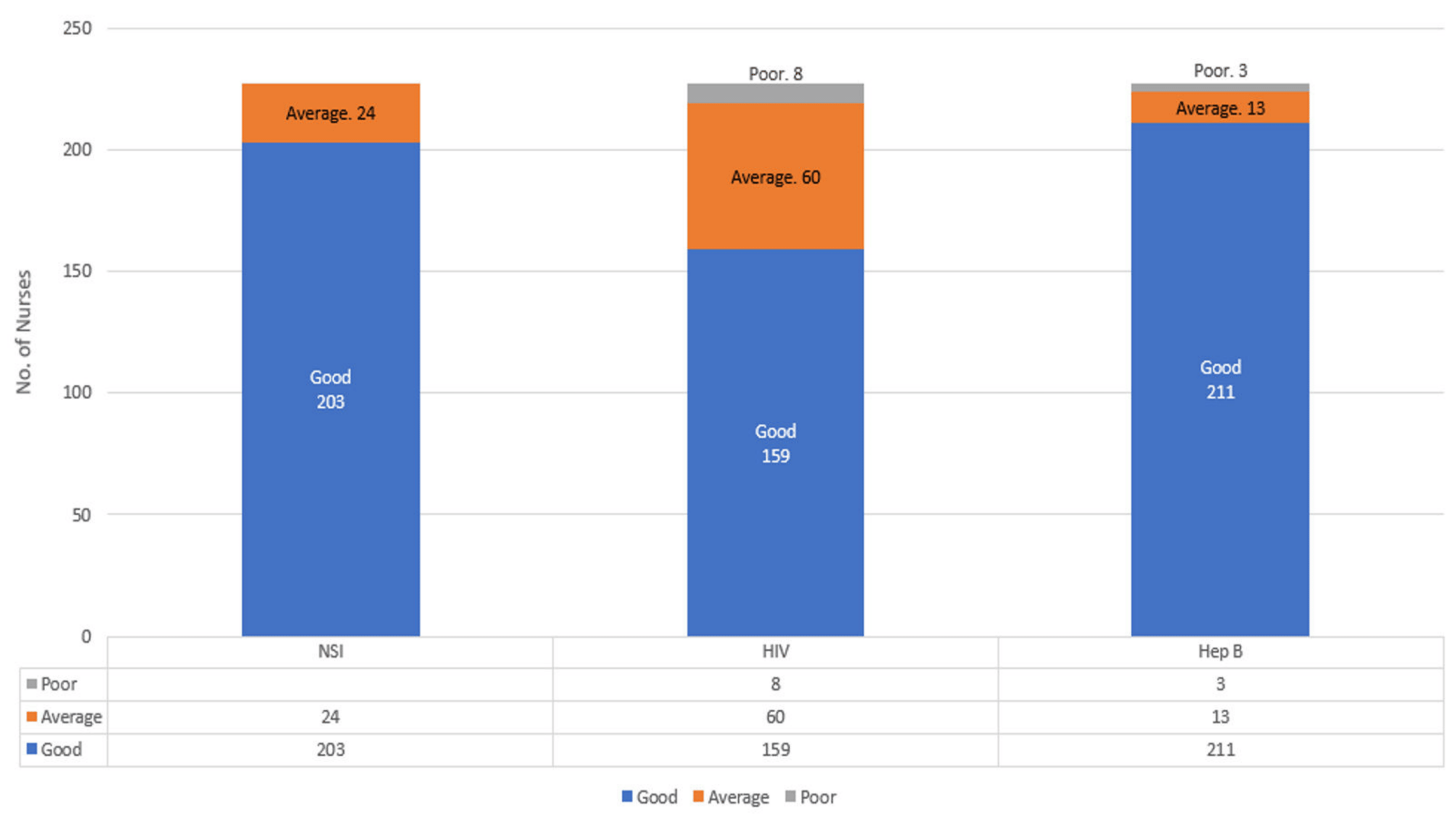
Knowledge levels for NSI, HIV, and Hepatitis B NSI: needlestick injury

The study included 227 nurses with a mean age of 27 ± 2.7 years. The majority were female (73.6%), had less than three years of experience (61%), and were unmarried (69.2%) (Table 1). The overall prevalence of NSI among participants was 40.5% (95% CI: 34.1% - 47.2%). NSI occurrence was not significantly influenced by age (p = 0.215), experience (p > 0.65), or marital status (p = 0.633). While males had higher NSI rates, the difference was not significant (p = 0.103), suggesting minimal impact of demographic factors (Table 2). The study revealed high overall knowledge levels, with 89.4% demonstrating a good understanding of NSI, 70% of HIV, and 93% of Hepatitis B. However, notable gaps persisted in HIV awareness, where 26.4% had average knowledge and 3.6% showed poor understanding (Figure 1). These findings highlight the need for targeted educational interventions to bridge knowledge deficits and strengthen prevention strategies.

**Table 1:** Basic demographic details of the study participants (N=227)

Characteristics		Value
Age in years (Mean±SD)		27±2.7
Gender (n (%))	Male	60 (26.4)
	Female	167 (73.6)
Total years of experience (Median (IQR))		2 (1.4 – 3)
Work experience (n (%))	<3 years	138 (61)
	≥3 years	89 (39)
Marital status (n (%))	Unmarried	157 (69.2)
	Married	70 (30.8)

**Table 2:** Comparison of needle stick injury (NSI) among different groups (N=227) ^Chi-squared/Fisher's exact test; *t-test and #Wilcoxon rank-sum test

Characteristics		NSI		Test statistic	p-value^^^
		Present (n=92)	Absent (n=135)		
Age in years (Mean±SD)		27.29±2.5	26.82±2.9	-1.242	0.215^*^
Gender (n (%))	Male	19 (32%)	41 (68%)	2.657	0.103
	Female	73 (43.7%)	94 (56.3%)		
Total years of experience (Median (IQR))		2 (1.5 – 3.35)	2 (1.3 – 3)	-0.432	0.665^#^
Work experience (n (%))	<3 years	56 (40.6%)	82(59.4%)	0.0004	0.984
	≥3 years	36 (40.4%)	53 (59.6%)		
Marital status (n (%))	Unmarried	62 (39.5%)	95 (60.5%)	0.227	0.633
	Married	30 (42.9%)	40 (57.1%)		

NSI incidence did not significantly differ based on knowledge levels of NSI, HIV, or Hepatitis B (p > 0.05). While minor variations were observed, they were not statistically significant, suggesting that knowledge alone may not directly influence NSI occurrence (Table 3). Demographic factors, such as age (p = 0.563), gender (p = 0.193), work experience (p = 0.794), and marital status (p = 0.112), did not significantly influence knowledge levels for NSI, HIV, or Hepatitis B (Table 4). These findings suggest that demographic characteristics did not have a meaningful impact on knowledge levels regarding NSI, HIV, or Hepatitis B, reinforcing the need for targeted education and intervention programs.

**Table 3:** Comparison of NSI among different knowledge levels for NSI, HIV, and Hepatitis B (N=227) ^Chi-squared/Fisher's exact test NSI: needlestick injury

Knowledge categories	NSI				HIV				Hepatitis B			
	NSI Category (n (%))											
	Present (n=92)	Absent (n=135)	Test Statistics	p-value^^^	Present (n=92)	Absent (n=135)	Test Statistics	p-value^^^	Present (n=92)	Absent (n=135)	Test Statistics	p-value^^^
Good	84 (91.3%)	119 (88.2%)	0.576	0.448	66 (71.7%)	93 (68.9%)	2.703	0.259	86 (93.5%)	125 (92.6%)	0.092	1.000
Average	8 (8.7%)	16 (11.8%)			25 (27.2%)	35 (25.9%)			5 (5.4%)	8 (5.9%)		
Poor	-	-			1 (1.1%)	7 (5.2%)			1 (1.1%)	2 (1.5%)		

**Table 4:** Comparison of knowledge regarding NSI, Hepatitis B, and HIV among different groups (N=227) ^Chi-squared/ Fisher's exact test; *ANOVA and #Kruskal Wallis test NSI: needlestick injury, ANOVA: analysis of variance

Characteristics		NSI knowledge				HIV knowledge					Hepatitis B knowledge				
		Good (n=203)	Average (n=24)	Test Statistics	p-value^^^	Good (n=159)	Average (n=60)	Poor (n=8)	Test Statistics	p-value^^^	Good (n=211)	Average (n=13)	Poor (n=3)	Test Statistics	p-value^^^
Age in years (Mean±SD)		27.05±2.8	26.71±2.6	-0.578	0.563^*^	26.83±2.53	27.61±3.11	26.12±3.94	2.19	0.114^*^	27±2.7	27.61±2.56	3±2.88	0.66	0.516^*^
Gender (n (%))	Male	51 (85%)	9 (15%)	1.691	0.193	41 (68.3%)	17 (28.3%)	2 (3.4%)	0.154	0.926	57 (95%)	3 (5%)	-	1.189	0.552
	Female	152 (91.1%)	15 (8.9%)			118 (70.6%)	43 (25.7 %)	6 (3.7%)			154 (92.2%)	10 (5.9%)	3 (1.8%)		
Total years of experience (Median (IQR))		2 (1.4 – 3)	2 (1.1 – 3.5)	0.091	0.927^#^	2 (1.5 – 3)	2 (1.25 – 4)	2 (0.7 – 2.5)	1.598	0.449^#^	2 (1.5 – 3)	2 (1.2 – 5)	2 (1 – 6)	0.075	0.963^#^
Work experience (n (%))	<3 years	124 (89.9%)	14 (10.1%)	0.068	0.794	97 (70.3)	35 (25.4%)	6 (4.3%)	0.833	0.659	127 (92.1%)	9 (6.5%)	2 (1.4%)	0.464	0.793
	≥3 years	79 (88.2%)	10 (11.8%)			62 (69.7%)	25 (28.1%)	2 (2.2%)			84 (94.4%)	4 (4.5%)	1 (1.1%)		
Marital status (n (%))	Unmarried	137 (87.3%)	20 (12.7%)	2.527	0.112	116 (73.9%)	35 (22.3%)	6 (3.8%)	4.499	0.105	145 (92.4%)	10 (6.4%)	2 (1.2%)	0.395	0.821
	Married	66 (94.3%)	4 (5.7%)			43 (61.4%)	25 (35.7%)	2 (2.9%)			66 (94.3%)	3 (4.4%)	1 (1.3%)		

A majority of participants (92.6%) expressed concern about the risk of NSI in their profession, and 97.7% believed NSI incidents were preventable. Most participants (96.8%) supported immediate reporting of sharp injuries, but common barriers to reporting included a lack of awareness and time constraints. Concern about acquiring HIV (93%) and Hepatitis B (93%) was widespread, yet only 10.2% and 11.1% felt uncomfortable managing patients with these infections, respectively. Support for mandatory testing before entering nursing was high (HIV: 84%, Hepatitis B: 89%), and most participants endorsed PEP for HIV (94.4%) and Hepatitis B (93%) (Table 5).

**Table 5:** Frequency distribution of attitude responses among the study participants regarding NSI, HIV, and Hepatitis B (N=227 participants) NSI: needlestick injury

Attitude Question	Response	Frequency (%)
1. I worry that since I am in the medical profession, I am at high risk of contracting a needle stick injury at any time in my career as compared to the general population. (n=219)	Yes	203 (92.6)
	No	16 (7.4)
2. Patient care is more important than the safety of nurses. (n=219)	Yes	32 (14.6)
	No	187 (85.4)
3. All sharp injuries at work should be reported immediately. (n=218)	Yes	211 (96.8)
	No	7 (3.2)
4. I think needle stick injury is preventable. (n=219)	Yes	214 (97.7)
	No	5 (2.3)
5. Sharp object waste should be disposed of by a professional company, not in domestic waste. (n=218)	Yes	208 (95.4)
	No	10 (4.6)
6. What do you think are the factors that can lead to a needlestick injury? (Multiple responses) (n=219)	Recapping	208 (95)
	Manipulating the needle inpatient	116 (53)
	Disposal related	169 (77)
	IV line related	92 (42)
	Clean up	61 (27.8)
	Collision with a fellow health worker	126 (57.5)
	Passing the needle after use	181 (82.6)
	Stress-related	69 (31)
	High patient load per nurse	164 (75)
7. What do you think are the reasons for the non-reporting of the needlestick injuries? (Multiple responses) (n=212)	I never knew that such incidents needed to be reported	77 (36)
	Being too busy managing patient time pressure	166 (78.3)
	Needlestick injury by a fresh needle rather than contaminated ones	166 (78.3)
	Not knowing how to report	98 (46)
	No need to worry if fully vaccinated	72 (34)
	Fear of stigma and embarrassment	79 (37.3)
8. I am concerned that since I am in the medical profession, I am at high risk of getting infected with a Hep B/HIV infection at work. (n=217)	Yes	202 (93)
	No	15 (7)
9. Hepatitis B vaccine is safe and effective. (n=218)	Yes	213 (97.7)
	No	5 (2.3)
10. Changing gloves during blood collection and tests is a waste of time. (n=218)	Yes	10 (4.6)
	No	208 (95.4)
11. I do not feel comfortable taking care of people with Hepatitis B. (n=216)	Yes	24 (11.1)
	No	192 (88.9)
12. I do not feel comfortable taking care of people with HIV. (n=216)	Yes	22 (10.2)
	No	194 (89.8)
13. All nurses should undergo mandatory HIV testing before joining the nursing profession. (n=213)	Yes	179 (84)
	No	34 (16)
14. All nurses should undergo mandatory Hepatitis B testing before joining nursing. (n=211)	Yes	188 (89)
	No	23 (11)
15. Post-exposure prophylaxis medications for HIV are available in our institution. (n=214)	Yes	196 (91.6)
	No	2 (0.9)
	Don’t know	16 (7.5)
16. Post-exposure prophylaxis medications for Hepatitis B are available at our institution. (n=214)	Yes	186 (86.9)
	No	9 (4.2)
	Don’t know	19 (8.9)
17. Do you recommend post-exposure prophylaxis for those who have been exposed to Hepatitis B? (n=216)	Yes	201 (93)
	No	15 (7)
18. Do you recommend post-exposure prophylaxis for those who have been exposed to HIV? (n=216)	Yes	204 (94.4)
	No	12 (5.6)

Among healthcare workers with NSI history, 61.8% had at least one incident, primarily superficial injuries (59.8%), with 65.2% reporting the event. However, 34.8% did not seek medical attention within two hours. Common responses included washing with soap and water (91%) and identifying the source patient (71.2%), yet only 31% received PEP. Hypodermic needles (24.1%) and lancets (20.7%) were the most frequently involved devices, with 41.2% of NSIs occurring during disposal or recapping, mainly in hospital wards (65.9%) and after joining the job (60%). While 90.9% did not acquire infections, 50% of those infected had Hepatitis B. Despite PEP being recommended, only 46.5% were offered it, and just 39.3% completed the regimen, often due to fear of side effects or assuming it was unnecessary. These findings highlight both strengths and critical gaps in NSI management, emphasizing the need for timely medical attention, improved PEP adherence, and stricter prevention strategies (Table 6).

**Table 6:** Practice followed regarding NSI, HIV, and Hepatitis B among those who had NSI (N=92 participants) ART: antiretroviral therapy

Practice Question	Response	N (%)
1. Number of needlestick injuries you have had in your career so far.	Once	57 (61.8)
	2-4 times	27 (29.4)
	5 or more times	4 (4.4)
	Yes, but I don’t remember the count	4 (4.4)
1.1 What was the injury type? (n=87)	Superficial (little or no bleeding)	52 (59.8)
	Moderate (skin punctured, some bleeding)	31 (35.6)
	Severe (deep stick/cut or profuse bleeding)	4 (4.6)
1.2 Did you report the needlestick injury?	Yes	60 (65.2)
	No	32 (34.8)
1.3 Did you receive medical attention within 2 h after injury	Yes	60 (65.2)
	No	32 (34.8)
1.4 What action was taken after injury - (Multiple-response question) (n=87)	Washed with soap and water	79 (91)
	Got tested for HIV, hepatitis B, and hepatitis C	55 (59.7)
	Identified the source patient	62 (71.2)
	Got post-exposure prophylaxis (PEP) when the source patient was unknown or tested positive for HIV & Hepatitis B	27 (31)
	Checked Anti-HBs titre	39 (42.3)
1.5 Device involved in the last incident. (n=87)	Intravenous (IV) cannula	16 (18.3)
	Butterfly needle	2 (2.3)
	Hypodermic needle	21 (24.1)
	Phlebotomy needle	14 (16.1)
	Lancets	18 (20.7)
	Razors	2 (2.3)
	Scissors	0
	Suture needle	7 (8.6)
	Others	7 (8.6)
1.6 At what point in time did the NSI occur? (n=85)	Before procedure	25 (29.4)
	During procedure	25 (29.4)
	While recapping the needle/ during disposal	35 (41.2)
1.7 Work area where the recent injury occurred? (n=85)	Outdoor patient room	4 (4.7)
	Hospital wards	56 (65.9)
	Emergency wards	7 (8.7)
	Intensive/Critical care unit	8 (9.4)
	Operating room/Recovery	7 (8.7)
	Others	3 (3.5)
1.8 When did the NSI occur? (n=85)	During graduation from nursing	32 (37.6)
	During post-graduation	2 (2.4)
	After joining a job	51 (60)
1.9 Did you ever get infected with any blood-borne infection after NSI? (n=88)	Yes	8 (9.1)
	No	80 (90.9)
1.10 If yes, which infection? (n=8)	HIV	3 (37.5)
	Hepatitis B	4 (50)
	Hepatitis C	1 (12.5)
1.11 Were you offered post-exposure prophylaxis? (n=28)	Yes	13 (46.5)
	No	15 (53.5)
1.12 Did you complete the post-exposure prophylaxis? (n=28)	Yes	11 (39.3)
	No	17 (60.7)
1.13 If No, what was the reason for discontinuation? (n=20)	Fear of adverse effects.	3 (15)
	Assuming that it was enough.	2 (10)
	Assuming that the drug was not effective.	1 (5)
	I had to purchase the Immunoglobulin/Vaccine/ART, which was costly.	0
	Other reasons	14 (70)

Unsafe practices such as recapping needles with two hands were significantly associated with NSI (p = 0.045). However, other practices, including bending needles before disposal, availability of safety containers, and proper disposal of sharp items, did not show significant associations with NSI occurrence. HBV vaccination status and the number of vaccine doses received were also not significantly correlated with NSI incidence (p = 0.827) (Table 7).

**Table 7:** Comparison of practices followed regarding NSI, HIV, and Hepatitis B between the NSI groups (N=227 participants) Chi-squared/Fisher's exact test

Practice Question	Response	NSI Present N=92	NSI Absent N=135	X^2^	p-value
		n (%)	n (%)		
2. Do you recap needles with 2 hands before disposal? (n=219)	Always	13 (59.1)	9 (40.9)	6.209	0.045
	Sometimes	17 (54.8)	14 (45.2)		
	No	62 (37.3)	104 (62.7)		
3. Do you bend needles before disposal? (n=216)	Always	19 (52.8)	17 (47.2)	2.393	0.302
	Sometimes	8 (47.1)	9 (52.9)		
	No	64 (39.3)	99 (60.7)		
4. Is the safety box/disposal container available at your workplace? (n=219)	Yes	87 (40.9)	126 (59.1)	1.602	0.236
	No	4 (66.7)	2 (33.3)		
5. Do you put sharp items into their assigned disposal container? (n=219)	Always	89 (41.4)	126 (58.6)	2.162	0.394
	Sometimes	1 (100)	0		
	No	2 (66.7)	1 (33.3)		
6. Do you change gloves for each patient during blood taking? (n=219)	Always	79 (41.4)	112 (58.6)	3.788	0.150
	Sometimes	5 (31.3)	11 (68.7)		
	No	8 (66.7)	4 (33.3)		
7. Have you been vaccinated against HBV? (n=220)	Yes	82 (40.6)	120 (59.4)	1.521	0.218
	No	10 (55.6)	8 (44.4)		
7.1 How many doses of HBV vaccine did you receive? (n=202)	1	4 (36.4)	7 (63.6)	0.458	0.827
	2	12 (36.4)	21 (63.6)		
	3	66 (41.8)	92 (58.2)		

To summarize, NSI prevalence was high (40.5%), but demographic factors did not significantly influence occurrence. Knowledge levels were high, but no significant relationship with NSI rates was observed. A strong attitude towards NSI prevention and reporting existed, but barriers to reporting persisted. Unsafe practices, particularly recapping needles, were a significant contributor to NSI. Despite available PEP, adherence remained low, highlighting a need for better post-exposure management strategies. These findings emphasize the importance of reinforcing safe needle practices, improving PEP adherence, and addressing barriers to reporting NSI incidents to reduce occupational risks among healthcare workers.

## Discussion

NSIs remain a major occupational hazard for healthcare workers (HCWs), posing risks of bloodborne infections, including Hepatitis B (HBV) and HIV. This study evaluated the knowledge, attitudes, and practices (KAP) related to NSI, HBV, and HIV among nurses in a tertiary care hospital in Nagpur, India.

The prevalence of NSI in this study was 40.5% (95% CI: 34.1% - 47.2%), comparable to global prevalence rates of 44.5% [13] and findings from Sudan (40%) [11]. However, it was higher than in Kerala, India (24.4%) [21] and Saudi Arabia (11.57%) [19] but lower than in Egypt (67.9%) [22]. The higher NSI prevalence observed in this study could be due to multiple factors, including nurses' workload, exposure to high-risk procedures, and adherence to preventive measures.

The mean age of participants was 27 years, which was lower than in other comparable studies [11,12,19,21] but higher than the study by Balegha et al. [23], which focused on nursing students. While Naidu et al. reported a higher NSI incidence among younger HCWs, in this study, age was not significantly associated with NSI prevalence (p = 0.215) [12]. The majority of participants were female (73.6%), which is consistent with the Sudanese study [11]. NSI was more prevalent among female nurses than male nurses, aligning with findings from Naidu et al. [12]. However, Goel et al. and Muralidhar et al. reported higher NSI prevalence in male HCWs [24,25]. Studies suggest that NSI risk decreases with experience [12]. In our study, most nurses had an average of two years of experience (range: 1.4-3 years), lower than in other studies [11,19], likely due to our hospital’s younger workforce. Consequently, NSI incidence did not significantly vary with experience. Balegha AN et al. observed that married nurses exhibited better practices than unmarried ones, attributing this to increased responsibility and guideline adherence post-marriage [23]. While 90% of nurses in their study were unmarried, our study had 69% unmarried nurses, yet NSI was more frequently reported among married nurses. Further research is needed to explore socio-demographic influences on NSI. The knowledge levels of participants were high, with 89.4% demonstrating good knowledge of NSI, 70% for HIV, and 93% for Hepatitis B. These results are comparable to studies from Sudan, Afghanistan, and Saudi Arabia [8,11,19]. However, despite high knowledge levels, NSI prevalence remained substantial, suggesting that knowledge alone may not directly reduce NSI risk. Unsafe behaviors persisted, with 24% of participants admitting to recapping needles, despite 64% of them experiencing NSI. This risk was lower than in Saudi Arabia, where 38.8% of HCWs engaged in recapping. In this study, 88% of participants recognized Hepatitis B as the most transmissible infection following NSI, a finding consistent with the Saudi Arabian study [19]. Additionally, 97% of nurses were aware of local guidelines for NSI management, aligning with findings from Sudan. However, only 52% knew that the correct immediate response to an NSI was washing the injury site with soap and water, which is lower than the Sudanese study, where 77.8% of participants identified the ideal method [11]. This discrepancy could be attributed to variations in training, years of experience, and exposure to standardized protocols across different healthcare systems. Regarding Hepatitis B transmission, 93% identified NSI as a risk, 77% acknowledged sexual transmission, and 87% recognized mother-to-child transmission, findings consistent with the Afghan study [8]. Knowledge of the vaccine schedule (86%) and post-exposure prophylaxis (93%) was higher than in Afghan [8] and Saudi studies [19]. However, only 69% knew Hepatitis B could lead to liver cancer, highlighting a gap in awareness that needs to be addressed.

Eighty percent (80%) of nurses correctly understood PEP for HIV, better than that shown in the study by Tshering et al. [9]. However, 8.4% falsely believed that HIV could be prevented by a vaccine, possibly due to misinformation prevalent in developing countries. Awareness of HIV PEP was high, with 96% knowing it exists, 86% learning about it during graduation, 64.7% identifying the correct initiation window, and 63.4% recognizing the 28-day regimen. These figures exceeded those reported in Bhutan [9], possibly due to differences in training curricula, exposure to standardized guidelines, or institutional emphasis on HIV PEP awareness.

Most nurses prioritized their own safety over patient care, with 92.6% expressing concern about an NSI risk. A majority supported immediate reporting of NSI, considered it preventable, and endorsed professional disposal of sharps, findings consistent with studies from Saudi Arabia and India [19,21]. Similarly, 93% perceived a high occupational risk of Hepatitis B and HIV, aligning with the Afghan study [8]. Despite this awareness, many nurses felt uncomfortable managing Hepatitis B or HIV-infected patients and advocated for PEP availability for NSI cases. The four main factors contributing to NSI included recapping needles (91.6%), passing used needles (79.6%), improper disposal (74.4%), and high patient load per nurse (72.2%). These differed from Alsabaani et al., where handling/passing needles was the leading cause, likely due to variations in training and workplace practices [19].

Barriers to NSI reporting included time constraints, fresh needle injuries, lack of reporting knowledge, and fear of stigma, consistent with Sudanese data and Alsabaani et al. [11,19]. However, in Madhavan et al., minor injuries were the most cited reason for non-reporting [21]. Additionally, many nurses supported mandatory HIV and Hepatitis B testing before entering the profession, mirroring findings from Afghanistan [8].

In our study, 61.8% of nurses experienced NSI once, while 33.8% had multiple NSIs, contrasting with the Saudi Arabian study, where 39.6% had a single NSI and 58.3% had multiple [19]. However, injury severity patterns were similar, with superficial injuries being the most common (59.8%) and severe injuries accounting for 4.6%. NSI reporting and medical attention rates were higher in our study, with 65.2% reporting to authorities and all receiving immediate care, whereas in Saudi Arabia, only 47.3% reported NSI, and 46.2% sought medical attention [19]. Post-injury practices varied, with 91% washing the injury site, 31% receiving PEP, 42.3% checking Anti-HBs titers, 71.2% identifying the source patient, and 59.7% getting tested for blood-borne infections, findings comparable to Saudi Arabian data. Hypodermic needles (24.1%) were the most common cause of NSI, followed by lancets (20.7%) and IV cannulas (18.3%), differing from Saudi Arabian data, where IV cannulas were predominant [19]. This may be due to frequent bedside glucose monitoring in our hospital. Most NSIs occurred during recapping or disposal (41.2%), primarily in hospital wards (65.9%), with 60% of cases reported post-employment. Among affected nurses, 9.1% developed blood-borne infections, mainly Hepatitis B (50%). Despite PEP availability, only 46.5% of affected nurses were offered it, and just 39.3% completed the full regimen, often due to fear of side effects. Recapping needles was significantly linked to NSI (p = 0.045), and unsafe practices like bending needles and not changing gloves were common, consistent with Afghan and Sudanese studies [8,11].

Hepatitis B vaccination coverage was 89%, higher than in Afghanistan (56.97%) but similar to Saudi Arabia [8,19]. Notably, nurses without NSI were more likely to have completed the full three-dose Hepatitis B vaccine, reinforcing its potential protective role.

Strengths

The strengths of this study are that it integrated findings from three distinct studies for a comprehensive analysis; focused exclusively on nurses, ensuring homogeneity in the study population; high response rates were achieved through a reminder system, minimizing bias; and categorization by NSI status allowed targeted knowledge analysis.

Limitations

The limitations of this study include: nurse rotations across departments could have influenced results; findings from a single tertiary care center may not have represented all nurses in the region; and a potential volunteer bias in data collection and non-participation due to shift schedules/workload constraints or declined consent may have impacted findings.

The study underscores the urgent need for infection control training, emphasizing safe handling and disposal of needles. High-risk behaviours, such as recapping needles, were significantly associated with NSI incidence. While knowledge levels were high, gaps in compliance and safe practices persist. Future research should focus on longitudinal studies assessing intervention effectiveness in reducing NSI rates and improving safety protocol compliance.

## Conclusions

The findings of this study provide a comprehensive assessment of the knowledge, attitudes, and practices (KAP) of nurses regarding needlestick injuries (NSI), HIV, and Hepatitis B in a tertiary care hospital. While the majority of nurses demonstrated high knowledge levels and positive attitudes, significant gaps were observed in safe practices, particularly needle recapping and adherence to post-exposure prophylaxis (PEP). Despite high awareness, unsafe practices persist, emphasizing the need for structured training programs, reinforcement of infection control policies, and continuous monitoring of compliance. The study highlights the urgent need for targeted interventions, including enhanced training on safe needle disposal, mandatory Hepatitis B vaccination policies, and routine PEP training and availability. Addressing these gaps can significantly enhance healthcare worker safety, reduce the occupational risks associated with NSI, and strengthen infection control measures. Future research should focus on longitudinal assessments of intervention effectiveness and policy-driven initiatives to further reduce the NSI incidence and improve compliance with safety protocols.

## Appendices

Appendix

Questionnaire for Assessing Knowledge, Attitudes, and Practices Regarding Needle Stick Injuries, HIV, and Hepatitis B Prevention

Section 1: Socio-demographic characteristics

**Table 8:** Section 1 (socio-demographic characteristics)

Sl. No	Question
1	Age
2	Gender
3	Workplace at Hospital
4	Years of experience
5	Marital status

Section 2: Knowledge of NSI, Hepatitis B, and HIV prevention

**Table 9:** Section 2 (knowledge of NSI, Hepatitis B, and HIV prevention)

Qn. No	Question	
1	What are the infections that can be most commonly transmitted through needlestick injury?	
	i	HIV
	ii	Cholera
	iii	Hepatitis B
	iv	Candida
	v	Hepatitis C
	vi	Influenza
	vii	Don’t know
	2	Recapping of the needle after performing nursing procedures is recommended to decrease the risk of needlestick injury.	
	A	Yes
	B	No
	C	Don’t know
	3	Dispose in a sharps container after performing procedures is recommended to decrease the risk of needlestick injury.	
	A	Yes
	B	No
	C	Don’t know
	4	How many doses of Hepatitis B vaccine are required for full protection from Hepatitis B?	
	A	0
	B	1
	C	2
	D	3
	E	4
	F	Don’t know
	5	After needlestick injury, out of HIV, Hepatitis B and Hepatitis C; Hepatitis B carries the greatest risk of transmission.	
	A	Yes
	B	No
	C	Don’t know
	6	HIV can be prevented by Vaccine.	
	A	Yes
	B	No
	C	Don’t know
	7	Are you aware of the procedure and guidelines to follow if you sustain a needlestick injury in your workplace?	
	A	Yes
	B	No
	C	Don’t know
	8	If you have a needlestick injury your immediate action will be-	
	A	to clean hands with sterile cotton
	B	to wash your hand with water only
	C	to wash your hands with soap and water
	D	Not sure
	9	Do you know that there is a Post exposure prophylaxis guideline available for prevention of Hepatitis B after a needlestick injury?	
	A	Yes
	B	No
	C	Not sure
	10	Do you know that there is a Post exposure prophylaxis guideline available for prevention of HIV after a needlestick injury?	
	A	Yes
	B	No
	C	Not sure
	11	Hepatitis B can be transmitted by unsafe sexual contact.	
	A	Yes
	B	No
	C	Don’t know
	12	Hepatitis B can be transmitted from mother to child during pregnancy.	
	A	Yes
	B	No
	C	Don’t know
	13	Hepatitis B can cause liver cirrhosis.	
	A	Yes
	B	No
	C	Don’t know
	14	Hepatitis B can cause Liver cancer.	
	A	Yes
	B	No
	C	Don’t know
	15	Hepatitis B Immunoglobulin can be used for post exposure prophylaxis.	
	A	Yes
	B	No
	C	Don’t know
	16	I was taught about post exposure prophylaxis for prevention of HIV during graduation.	
	A	Yes
	B	No
	C	Don’t know
	17	I was taught about post exposure prophylaxis for prevention of Hepatitis B during graduation.	
	A	Yes
	B	No
	C	Don’t know
	18	Meaning of Post exposure prophylaxis for HIV-	
	A	Medical help given to prevent occupational transmission of HIV before exposure.
	B	Medical help given to clients after the transmission of HIV.
	C	Medical help given to prevent the occupational transmission of HIV after exposure.
	D	Psychological support given HIV positive people
	E	Not sure
	19	Time for Post exposure prophylaxis initiation for HIV-	
	A	When the source patient is at high risk for HIV
	B	When the patient is known to be HIV positive.
	C	When the HIV status of the source is unknown
	D	For any needle stick injury in the work place
	E	Not sure
	20	Preferable time to take Post exposure prophylaxis for HIV-	
	A	Within an hour
	B	Within 6 hours of exposure
	C	Within 12 hours of exposure
	D	Within 72 hours of exposure
	E	Don’t know
	21	Duration to take Post exposure prophylaxis for HIV-	
	A	7 days
	B	14 days
	C	28 days
	D	40 days
	E	Don’t know

Section 3: Attitude of nurses toward NSI and the prevention of HIV and Hepatitis B

**Table 10:** Section 3 (Attitude of nurses toward NSI and prevention of HIV and Hepatitis B)

Qn. No	Questions	
1	I am worried about having needle stick injury.	
	A	Yes
	B	No
	2	Patient care is more important than the safety of nurses.	
	A	Yes
	B	No
	3	All sharp injuries at work should be reported immediately.	
	A	Yes
	B	No
	4	I think needle stick injury is preventable.	
	A	Yes
	B	No
	5	Sharp objects waste should be disposed of by a professional company not in domestic waste.	
	A	Yes
	B	No
	6	What do you think are the factors that can lead to a needlestick injury? (Select best 5 answers)	
	A	Recapping
	B	Manipulating needle in a patient
	C	Disposal related
	D	IV line related
	E	Clean up
	F	Collision with fellow health care worker carrying a needle
	G	Passing needle after use
	H	Stress related
	I	High patient load per nurse
	7	What do you think are the reasons for non-reporting of the needlestick injuries? (Select best 4)	
	A	Never knew that such incidences are need to be reported
	B	Being too busy with managing patients-Time pressure
	C	Needlestick injury by fresh needle rather than contaminated ones
	D	Not knowing how to report
	E	No need to worry if fully vaccinated
	F	Fear of stigma and embarrassment
	8	I am concerned about Hepatitis B infection at work.	
	A	Yes
	B	No
	9	I am concerned about HIV infection at work.	
	A	Yes
	B	No
	10	Hepatitis B vaccine is safe and effective.	
	A	Yes
	B	No
	11	Changing of gloves during blood collection and tests is waste of time.	
	A	Yes
	B	No
	12	I do not feel comfortable to take care of people with Hepatitis B patients	
	A	Yes
	B	No
	13	I do not feel comfortable to take care of people with HIV patients.	
	A	Yes
	B	No
	14	All nurses should undergo mandatory HIV testing before joining nursing profession.	
	A	Yes
	B	No
	15	All nurses should undergo mandatory Hepatitis B testing before joining nursing profession.	
	A	Yes
	B	No
	16	Post exposure prophylaxis medications for HIV are available in our institution.	
	A	Yes
	B	No
	C	Don’t know
	17	Post exposure prophylaxis medications for Hepatitis B are available in our institution.	
	A	Yes
	B	No
	C	Don’t know
	18	Do you recommend Post exposure prophylaxis for those who had been exposed to Hepatitis B?	
	A	Yes
	B	No
	19	Do you recommend Post exposure prophylaxis for those who had been exposed to HIV?	
	A	Yes
	B	No

Section 4: Practices of nurses toward NSI and the prevention of HIV and Hepatitis B

**Table 11:** Section 4 (Practices of nurses toward NSI and the prevention of HIV and Hepatitis B)

Qn. No	Questions	
1	Number of Needlestick injuries you had in your career so far.	
	A	Zero
	B	Once
	C	2-4 times
	D	5 or more times
	E	Don’t remember
	1.1	What was the injury type?	
	A	Superficial (little or no bleeding)
	B	Moderate (skin punctured, some bleeding)
	C	Severe (deep stick/cut, or profuse bleeding)
	1.2	Did you report the Needlestick injury?	
	A	Yes
	B	No
	1.3	Did you receive medical attention within 2 h after injury?	
	A	Yes
	B	No
	1.4	What action was taken after the injury? - (Multiple-response question)	
	A	Washed with soap and water
	B	Got tested for HIV, hepatitis B, and hepatitis C
	C	Identified the source patient
	D	Got post-exposure prophylaxis (PEP) when the source patient was unknown or tests positive for HIV & Hepatitis B
	E	Checked Anti-HBs titer
	1.5	Device involved in the last incident.	
	A	Intravenous (IV) cannula
	B	Butterfly needle
	C	Hypodermic needle
	D	Phlebotomy needle
	E	Lancets
	F	Razors
	G	Scissors
	H	Suture needles
	I	Others
	1.6	At what point of time did the NSI occur?	
	A	Before procedure
	B	During procedure
	C	While recapping the needle/during disposal
	1.7	Work area where recent injury occurred?	
	A	Outdoor patient room
	B	Hospital wards
	C	Emergency wards
	D	Intensive/Critical care unit
	E	Operating room/Recovery
	F	Others......................................
	1.8	When did the NSI occur?	
	A	During graduation for nursing
	B	During post-graduation
	C	After joining job
	1.9	Did you ever got infected with any blood borne infection after NSI?	
A		Yes
B		No
1.9.1		If yes, which infection?	
	A	HIV
	B	Hepatitis B
	C	Hepatitis C
	D	Others
	1.10	Were you offered post-exposure prophylaxis?	
	A	Yes
	B	No
	1.11	Did you complete the post-exposure prophylaxis?	
	A	Yes
	B	No
	1.11.1	If no, what was the reason for discontinuation?	
	A	Fear of adverse effects
	B	Assuming that it was enough
	C	Assuming that the drug was not effective
	D	I had to purchase the Immunoglobulin/vaccine/ART that was costly
	E	Other reasons: (mention)
	2	Do you recap needles with 2 hands before disposal?	
	A	Yes
	B	No
	3	Do you bend needles before disposal?	
	A	Yes
	B	No
	4	Is the safety box/disposal container usually available?	
	A	Yes
	B	No
	5	Do you always put sharp items into their assigned disposal container?	
	A	Yes
	B	No
	6	Do you always change gloves for each patient during blood taking?	
	A	Yes
	B	No
	7	Have you got vaccinated against HBV?	
	A	Yes
	B	No
	7.1	How many doses of HBV vaccine did you receive?	
	A	1
	B	2
	C	3
